# Perceived severity and benefits show a preliminary conditional association with kidney-protective behaviors in type 2 diabetes with early kidney function decline: an exploratory health belief model analysis

**DOI:** 10.1080/0886022X.2026.2717464

**Published:** 2026-10-15

**Authors:** Chorphaka Fakkaew, Sarunya Koositamongkol, Sarinrut Sriprasong, Wiwat Chancharoenthana

**Affiliations:** ^a^ Department of Medical Nursing, Faculty of Nursing, Mahidol University, Bangkok, Thailand; ^b^ Department of Clinical Tropical Medicine, Faculty of Tropical Medicine, Mahidol University, Bangkok, Thailand; ^c^ Tropical Immunology and Translational Research Unit (TITRU), Department of Clinical Tropical Medicine, Faculty of Tropical Medicine, Mahidol University, Bangkok, Thailand

**Keywords:** Health behavior, type 2 diabetes, early chronic kidney disease, perceived severity, perceived benefits, Health Belief Model

## Abstract

Diabetic kidney disease (DKD) is a leading cause of chronic kidney disease (CKD) and end-stage kidney disease, and early-stage CKD represents a critical window to slow progression through self-management behaviors. The Health Belief Model (HBM) posits that perceived severity, benefits, barriers, and self-efficacy shape health behaviors; however, whether these constructs operate independently or interact in adults with type 2 diabetes and early kidney function decline remains unclear. This cross-sectional study examined four selected HBM constructs—perceived severity, perceived benefits, perceived barriers, and self-efficacy—both individually and through their interaction, as predictors of self-reported kidney-protective health behaviors, assessed simultaneously *via* questionnaires, among 123 adults with type 2 diabetes and early-stage CKD or at high risk for CKD (eGFR 45–89 mL/min/1.73m^2^; G3a, *n* = 26; G2 without albuminuria confirmation, *n* = 97) at a tertiary hospital in Bangkok. The four HBM constructs did not independently predict behaviors (*F* = 2.161, *p* = 0.078). Simple slopes analysis revealed that perceived severity was significantly associated with health behaviors at high perceived benefits (+1 SD: slope = 0.187, *p* = 0.041) but not at low levels (−1 SD: *p* = 0.524). These preliminary, hypothesis-generating findings suggest a potential conditional association between perceived severity and perceived benefits; however, the interaction term did not reach statistical significance (*p* = 0.289). Educational strategies targeting both disease severity awareness and self-management benefits may warrant further investigation. Longitudinal and adequately powered studies are needed to confirm these interaction patterns and establish directionality.

## LAY SUMMARY (PLAIN LANGUAGE)

Diabetic kidney disease is a common complication of type 2 diabetes that can gradually worsen without early management. We studied 123 Thai adults with type 2 diabetes and early-stage kidney disease to understand what motivates protective behaviors such as healthy eating, exercise, and medication adherence. Using the Health Belief Model, we found that no single factor—believing the disease is serious, believing that self-care helps, perceiving barriers, or feeling confident—predicted protective behaviors on its own. However, patients who both recognized their kidney disease as serious and believed that self-management would help tended to engage in more protective behaviors in this preliminary analysis. Neither belief alone was sufficient. These preliminary findings suggest that health education should address both the seriousness of the condition and the practical benefits of taking action.

## KEY MESSAGES

1. **What is known:** The Health Belief Model posits that perceived severity, benefits, barriers, and self-efficacy independently predict health behaviors, yet their joint and interactive roles in early diabetic kidney disease remain inadequately explored.
2. **This study adds:** In 123 adults with type 2 diabetes and early kidney function decline, no individual Health Belief Model construct independently predicted kidney-protective behaviors; however, exploratory simple slope analysis revealed that perceived severity was conditionally associated with protective behaviors only when perceived benefits were high (p = 0.041), suggesting a preliminary interaction pattern.
3. **Potential impact:** These hypothesis-generating findings suggest that educational interventions combining disease severity awareness with benefit-oriented self-management messaging may warrant evaluation in adequately powered longitudinal studies.

## GRAPHICAL ABSTRACT

**Figure:**
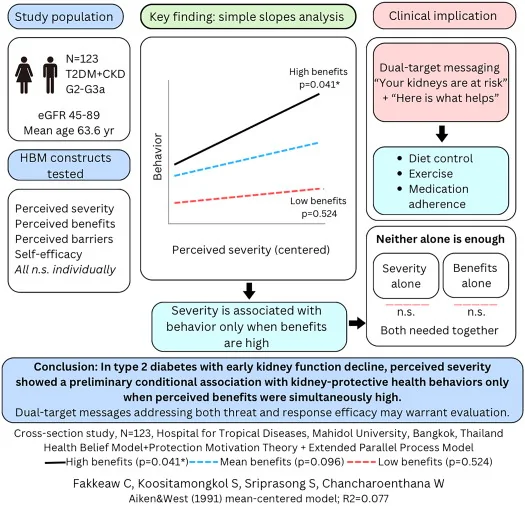

## Introduction

Diabetic kidney disease (DKD) is a prevalent complication of diabetes and a leading cause of chronic kidney disease (CKD) worldwide [1,2]. In Thailand, diabetes remains highly prevalent, and glycemic control is often suboptimal at the population level [3,4]. Early-stage CKD (G2–G3a) is typically asymptomatic and can delay diagnosis and preventive care, allowing progression to end-stage kidney disease (ESKD) [5]. However, guideline-concordant management, when combined with sustained self-care behaviors such as dietary control and medication adherence, can significantly slow this decline [6]. Understanding what motivates patients to adopt these self-reported protective behaviors during the critical early-stage window is therefore essential for designing effective interventions. Notably, the present study examines self-management behaviors as the outcome rather than clinical endpoints such as CKD progression or metabolic control.

The Health Belief Model (HBM) offers a well-established theoretical framework for understanding health behavior adoption. The model posits that individuals are more likely to engage in health-protective actions when they perceive the condition as severe (perceived severity), believe that taking action will reduce the threat (perceived benefits), encounter fewer obstacles to action (perceived barriers), and feel confident in their ability to perform the behavior (self-efficacy) [7,8]. While studies have traditionally examined these constructs as independent predictors, emerging evidence suggests that the relationships among HBM components may be more complex, with certain factors potentially working in combination rather than in isolation to influence behavior. This study focused on four HBM-related constructs, including perceived severity, perceived benefits, perceived barriers, and self-efficacy. Perceived susceptibility and cues to action were not included in the present analysis and should be examined in future studies to provide a more complete test of the HBM.

Contemporary behavioral theories provide a stronger conceptual basis for expecting interactive effects. Protection Motivation Theory (PMT) explicitly proposes that threat appraisal (perceived severity and vulnerability) and coping appraisal (response efficacy and self-efficacy) combine to influence protection motivation, with the highest level of motivation occurring when both appraisals are elevated [9]. Similarly, the Extended Parallel Process Model (EPPM) argues that high perceived threat coupled with high perceived efficacy leads to adaptive protective behaviors (danger control), whereas high threat without efficacy may lead to maladaptive responses such as denial or avoidance (fear control) [10]. These frameworks suggest that perceived severity (threat appraisal) and perceived benefits—which can be viewed as a proxy for response efficacy—may interact to predict health behavior rather than operating as independent additive predictors.

In early-stage DKD or CKD high-risk populations, patients are typically asymptomatic; therefore, perceived severity alone may be insufficient to promote self-management if patients do not believe that kidney-protective behaviors have practical benefits. Conversely, when patients recognize the seriousness of kidney disease, perceived benefits may be more likely to translate into concrete health behaviors. Therefore, the interaction between perceived severity and perceived benefits may provide a more clinically meaningful explanation of kidney-protective behaviors than either construct alone.

Although the HBM has been applied to various chronic illnesses, its ability to predict kidney-protective behaviors during the asymptomatic window of early DKD remains inadequately explored [11–13]. Specifically, it is unclear whether HBM constructs operate independently or interact in this population. Given that early-stage CKD patients often lack symptom-driven cues to action, understanding how perceptions of severity and the perceived value of self-management interact to shape behavior may be particularly important for tailoring interventions.

Therefore, this study aimed to examine the predictive value of HBM constructs—both individually and in combination—for kidney-protective health behaviors in Thai adults with type 2 diabetes and early-stage CKD. Grounded in the HBM and informed by the PMT and EPPM frameworks, we hypothesized that in addition to main effects, interactions among HBM constructs—particularly between perceived severity and perceived benefits, and potentially involving perceived barriers and self-efficacy—may play a role in predicting behavior in this population, with important implications for clinical practice and patient education.

## Methods

### Design, setting, and participants

We conducted a predictive correlational study at a university-hospital outpatient diabetes-nephrology clinic of the Hospital for Tropical Diseases, Mahidol University (Bangkok), between March and September 2024. Adults with type 2 diabetes and an eGFR of 45–89 mL/min/1.73 m^2^ were enrolled. This eGFR range corresponds to CKD stages G2–G3a, which is consistent with the Kidney Disease Improving Global Outcomes (KDIGO) framework. We note that albuminuria data were not systematically collected in this study; therefore, for participants with an eGFR of 60–89 (G2), a definitive CKD diagnosis per the KDIGO criteria could not be confirmed. These individuals are classified as being at high risk for CKD, aligning with the proactive approach of the KDIGO 2024 guidelines [14]. The distribution by CKD stage was as follows: G2 (eGFR 60–89), *n* = 97 (78.9%), and G3a (eGFR 45–59), *n* = 26 (21.1%).

The inclusion criteria included age ≥18 years; Thai language proficiency; a Mini-Cog score ≥3 if the patient was ≥60 years for cognitive impairment screening [15,16]; and an Eastern Cooperative Oncology Group (ECOG) performance status of 0–1 [17]. The exclusion criteria included congenital kidney disease, other primary kidney diseases, pregnancy, cancer, or clinically significant heart disease in the prior 6 months. Participants were recruited consecutively during clinic sessions attended by the research team. All HBM construct scores and health behavior outcomes were collected simultaneously *via* self-administered questionnaires at a single time point; therefore, the study cannot establish temporal precedence or directionality among the variables. The total number of patients screened was not formally recorded as a study-level variable; this represents a reporting limitation acknowledged in accordance with STROBE guidance.

A total of 123 participants met all inclusion criteria, provided informed consent, and completed the questionnaire battery on site. Two participants provided partially incomplete responses but were retained in the analysis, as no item-level missing data affected the main study variables. The sample size (*n* = 123) was estimated using G*Power 3.1 based on prior effect sizes (f^2^ = 0.169, α = 0.05, power = 0.95) for a four-predictor main effects model. We acknowledge that interaction effects typically require larger samples than main effects and that the sample may have been underpowered for interaction detection; this is noted as a limitation. The study protocol was approved by the Faculty of Nursing, Mahidol University, and the collaborating hospital IRB (MU-MOU CoA No. IRB-NS2023/743.2201) and conducted in accordance with the Declaration of Helsinki. Written informed consent was obtained from all participants. Reporting follows the STROBE checklist for cross-sectional studies (Supplementary Table S1).

### Measures

**Screening.** Cognition in participants aged ≥60 years was screened with the Mini-Cog (Thai version, 3-word recall + clock drawing) [15,16]. Performance status was assessed using the ECOG scale [17]. Data collection included demographic and clinical data, such as age, sex, education, income, diabetes duration, body mass index (BMI), estimated glomerular filtration rate (eGFR) calculated using the CKD-EPI Equation [18,19], and HbA1c, blood pressure, comorbidities, medications, and herb/supplement use.

**Instrument development and validation.** All HBM scales and the health behavior questionnaire were developed by the research team specifically for this study in Thai, following established scale development guidelines [20]. Item generation was informed by a comprehensive literature review of HBM applications in CKD and diabetes populations and consultation with clinical experts in nephrology nursing. As the instruments were developed in Thai by native Thai-speaking researchers for a Thai-speaking population, no translation or back-translation procedures were required.

Content validity was established through review by a five-member expert panel comprising nephrologists, diabetes nurse educators, and a behavioral health specialist. The item-level content validity index (I-CVI) ranged from 0.70 to 1.00, and the scale-level CVI (S-CVI) ranged from 0.70 to 1.00 [20]. Pilot testing was conducted with 30 patients meeting the same eligibility criteria (not included in the main analysis) to assess item clarity (mean completion time: 35 min), internal consistency (Cronbach’s α = 0.75–0.97), and item-total correlations (all > 0.30 except one reverse-scored item that showed a marginally low value; this item was retained based on its conceptual relevance to the construct and because its removal did not substantively alter internal consistency). For the self-efficacy scale (VAS format), test–retest reliability was assessed using the intraclass correlation coefficient (ICC ≥ 0.75, indicating good temporal stability) [21]. We acknowledge that formal factorial validation (EFA/CFA) and test–retest reliability for the Likert-based scales were not conducted, which is noted as a limitation. All individual scale items are presented in Supplementary Table S2. Classification cutoff points for low, moderate, and high categories were determined using the mean ± SD method.

**HBM constructs.** Perceived severity (10 items, 5-point Likert, range 10–50; α = 0.973), perceived benefits (8 items, 4-point, range 8–32; α = 0.950), and perceived barriers (9 items, 4-point, range 9–36; α = 0.946) were measured with Likert-type scales. Perceived self-efficacy was assessed with 5 items on a 0–100 VAS (mean score; α = 0.766), chosen to capture finer gradations of confidence per Bandura’s recommendation [8]. The difference in scale format between the VAS-based self-efficacy measure and the Likert-based constructs may have influenced the pattern of associations.

**Health behaviors.** Health behaviors were assessed using 15 items across five domains (diet, exercise, medication-taking, smoking avoidance, and stress management) on a 5-point Likert scale (7 positively worded, 8 negatively worded reverse-scored; α = 0.754). The composite total score was used as the primary outcome to provide a comprehensive assessment of overall kidney-protective lifestyle, which is consistent with clinical guidelines emphasizing multidomain self-management [6,14]. Domain-specific subscale scores were examined in exploratory analyses (Supplementary Table S3). The content validity of all the instruments was established by five experts (the item-CVI and scale-CVI ranged from 0.70 to 1.00) [20–22].

### Procedures

Eligible patients were approached at the outpatient diabetes-nephrology clinic, which serves as both a tertiary referral center and a primary care provider for patients with diabetes and CKD in the Bangkok metropolitan area. This setting was chosen to ensure access to clinically confirmed eGFR values and medical records for CKD staging verification [14]. The questionnaires were self-administered, with read-aloud support provided when needed. The completion time was approximately 30–45 min. Trained study personnel obtained consent and administered instruments in a private area. Participants were informed that they could terminate their participation at any time without any impact on their treatment or care.

### Statistical analysis

All analyses were performed using SPSS Statistics version 18.0 and Python (Statsmodels), with a two-sided significance level of α = 0.05. Descriptive statistics summarized sample characteristics and scale scores. Pearson product–moment correlation coefficients with 95% confidence intervals were used to examine the bivariate associations among all study variables.

A two-step analytical approach was employed to test our hypotheses. First, multiple linear regression using the enter method was used to determine whether the four HBM constructs (perceived severity, perceived benefits, perceived barriers, and self-efficacy) independently predicted health behavior scores. Regression assumptions, including linearity, independence of errors, homoscedasticity, and normality of residuals, were verified prior to analysis [21]. Variance Inflation Factors (VIF) were examined to assess multicollinearity, with values less than 2.0 considered acceptable.

Second, following the recommendations of Aiken and West (1991), perceived severity and perceived benefits were mean centered prior to computing the interaction term [23]. The interaction model included both mean-centered main effects (severity, benefits) and their product term, with perceived barriers and self-efficacy retained as covariates. Simple slopes of the severity–behavior relationship were tested at low (−1 SD), mean, and high (+1 SD) levels of perceived benefits. VIF values for the three mean-centered terms (severity, benefits, and their interaction) remained below 1.4 after mean centering; the covariates (perceived barriers and self-efficacy) showed higher VIF values (8.2 and 8.4, respectively), likely reflecting shared variance with other predictors, though this does not affect the focal interaction estimates.

Third, to assess robustness, the model was re-estimated with adjustment for six clinically relevant covariates: age, education level (dichotomized as below vs. at or above a bachelor’s degree), duration of diabetes (recoded to midpoint years), HbA1c level, eGFR, and number of comorbidities. A sensitivity analysis restricted to the G3a subgroup was also conducted. Exploratory domain-specific analyses and tests for nonlinear and moderating effects of self-efficacy were performed. All regression coefficients are reported as standardized β values with 95% confidence intervals.

## Results

### Participant characteristics

The baseline characteristics of the 123 participants are summarized in Table 1. The sample was predominantly female (53.7%), with a mean age of 63.59 years (SD = 10.97). Most participants were aged over 60 years (61.8%), and most had completed at least a bachelor’s degree (54.5%). Most participants had at least one comorbidity (93.5%), with 51.2% having two comorbidities and 38.2% having one. The mean duration of diabetes was substantial, with 38.2% having diabetes for more than 10 years and 37.4% having diabetes for 1–5 years. The mean HbA1c was 7.02% (SD = 1.35), indicating suboptimal glycemic control. The mean eGFR was 71.64 mL/min/1.73 m^2^ (SD = 12.88), confirming early-stage CKD (G2–G3a). The distribution by CKD stage was as follows: G2 (eGFR 60–89), *n* = 97 (78.9%), and G3a (eGFR 45–59), *n* = 26 (21.1%).

**Table 1.** Baseline characteristics of study participants (*N* = 123).

Characteristic	Value
*Demographic*	
Sex, n (%)	
Female	66 (53.7)
Male	57 (46.3)
Age (years), Mean (SD)	63.59 (10.97)
Age Group, n (%)	
31–40	6 (4.9)
41–50	8 (6.5)
51–60	33 (26.8)
> 60	76 (61.8)
Education, n (%)	
Primary School	12 (9.8)
Secondary School	21 (17.0)
Vocational Certificate	5 (4.1)
Diploma/associate degree	18 (14.6)
Bachelor’s Degree	46 (37.4)
Master’s Degree or higher	21 (17.1)
*Clinical*	
Duration of Diabetes, n (%)	
< 1 year	11 (9.0)
1–5 years	46 (37.4)
6–10 years	19 (15.4)
> 10 years	47 (38.2)
Number of Comorbidities, n (%)	
0	8 (6.5)
1	47 (38.2)
2	63 (51.2)
3	5 (4.1)
HbA1c (%), Mean (SD)	7.02 (1.35)
Systolic BP (mmHg), Mean (SD)	135.87 (16.21)
Diastolic BP (mmHg), Mean (SD)	77.14 (11.28)
BMI (kg/m^2^), Mean (SD)	27.23 (5.29)
eGFR (mL/min/1.73 m^2^), Mean (SD)	71.64 (12.88)

### Descriptive Statistics for HBM constructs and health behaviors

The descriptive statistics for the HBM constructs and health behaviors are presented in Table 2. Overall, kidney-protective health behaviors were moderate (mean = 49.72, SD = 8.13), with most participants (67.5%) falling into this category, followed by 17.1% at high levels and 15.4% at low levels. The perceived severity of diabetic kidney disease was moderate (mean = 33.08, SD = 9.94), with 64.2% of participants reporting moderate levels. The perceived benefits were also moderate (mean = 25.12, SD = 4.97). The perceived barriers were relatively low to moderate (mean = 16.99, SD = 5.20). Perceived self-efficacy was moderate (mean = 78.36, SD = 12.02).

**Table 2.** Descriptive Statistics for HBM constructs and health behaviors (*N* = 123).

Variable	Mean (SD)	Possible Range	Low, *n* (%)	Moderate, *n* (%)	High, *n* (%)
Health behaviors	49.72 (8.13)	15–75	19 (15.4)	83 (67.5)	21 (17.1)
Perceived severity	33.08 (9.94)	10–50	23 (18.7)	79 (64.2)	21 (17.1)
Perceived benefits	25.12 (4.97)	8–32	16 (13.0)	82 (66.7)	25 (20.3)
Perceived barriers	16.99 (5.20)	9–36	21 (17.1)	85 (69.1)	17 (13.8)
Self-efficacy	78.36 (12.02)	0–100	15 (12.2)	98 (79.7)	10 (8.1)

### Bivariate correlations

Pearson correlation analysis was used to examine the relationships among the HBM constructs and health behaviors (Table 3). Perceived severity was significantly and positively correlated with health behaviors (*r* = 0.214, 95% CI [0.038, 0.377], *p* = 0.018), as were perceived benefits (*r* = 0.180, 95% CI [0.003, 0.346], *p* = 0.047). In contrast, perceived barriers (r = −0.011, *p* = 0.907) and self-efficacy (*r* = 0.098, *p* = 0.280) were not significantly correlated with health behaviors. A moderate positive correlation was observed between perceived severity and perceived benefits (*r* = 0.463, 95% CI [0.311, 0.591], *p* < 0.001).

**Table 3.** Pearson correlation coefficients with 95% confidence intervals (*N* = 123).

Variable	Severity	Benefits	Barriers	Self-efficacy	Health behaviors
Severity	1	0.463** [0.311, 0.591]	0.139 [−0.039, 0.309]	−0.095 [−0.267, 0.084]	0.214* [0.038, 0.377]
Benefits		1	0.063 [−0.115, 0.237]	−0.039 [−0.214, 0.139]	0.180* [0.003, 0.346]
Barriers			1	−0.233** [−0.394, −0.058]	−0.011 [−0.187, 0.167]
Self-efficacy				1	0.098 [−0.080, 0.270]
Health behaviors					1

* *p* < 0.05, **p < 0.01. Values in brackets are 95% confidence intervals.

### Predictive factors for health behaviors

#### Main effects model (enter multiple regression)

Multiple linear regression using the enter method was conducted to examine whether the four HBM constructs independently predicted health behaviors. As shown in Table 4, the overall model was not statistically significant (*F* = 2.161, *p* = 0.078), accounting for only 6.8% of the variance in health behaviors (R^2^ = 0.068, adjusted R^2^ = 0.037). None of the individual predictors reached significance: perceived severity (β = 0.180, 95% CI [−0.020, 0.379], *p* = 0.078), perceived benefits (β = 0.102, 95% CI [−0.096, 0.300], *p* = 0.311), perceived barriers (β = −0.015, *p* = 0.869), or self-efficacy (β = 0.116, *p* = 0.209).

**Table 4.** Multiple linear regression (enter method) for predicting health behaviors (*N* = 123).

Predictor	b	SE	β	95% CI for β	*t*	*p* value
Constant	26.159	6.274	—	—	4.170	<0.001
Perceived severity	0.131	0.074	0.180	[−0.020, 0.379]	1.776	0.078
Perceived benefits	0.149	0.146	0.102	[−0.096, 0.300]	1.017	0.311
Perceived barriers	−0.021	0.129	−0.015	[−0.197, 0.166]	−0.165	0.869
Self-efficacy	0.070	0.055	0.116	[−0.065, 0.296]	1.262	0.209

Note: R^2^ = 0.068, Adjusted R^2^ = 0.037, *F* = 2.161, *p* = 0.078.

#### Mean-centered interaction model (Aiken & West, 1991)

Following Aiken and West’s (1991) framework [23], perceived severity and perceived benefits were mean centered, and the interaction model included both main effects and the interaction term (Table 5, Figure 1). The overall model explained 7.7% of the variance (R^2^ = 0.077; adjusted R^2^ = 0.038; *F* = 1.958; *p* = 0.090). The mean-centered interaction term (severity × benefits) was β = 0.095 (95% CI [−0.081, 0.271], *p* = 0.289). Perceived severity showed a trend toward significance (β = 0.171, 95% CI [−0.030, 0.371], *p* = 0.096). VIF values for the three mean-centered terms were below 1.4, confirming adequate multicollinearity control for the focal interaction estimates (Table 5).

**Figure 1.**
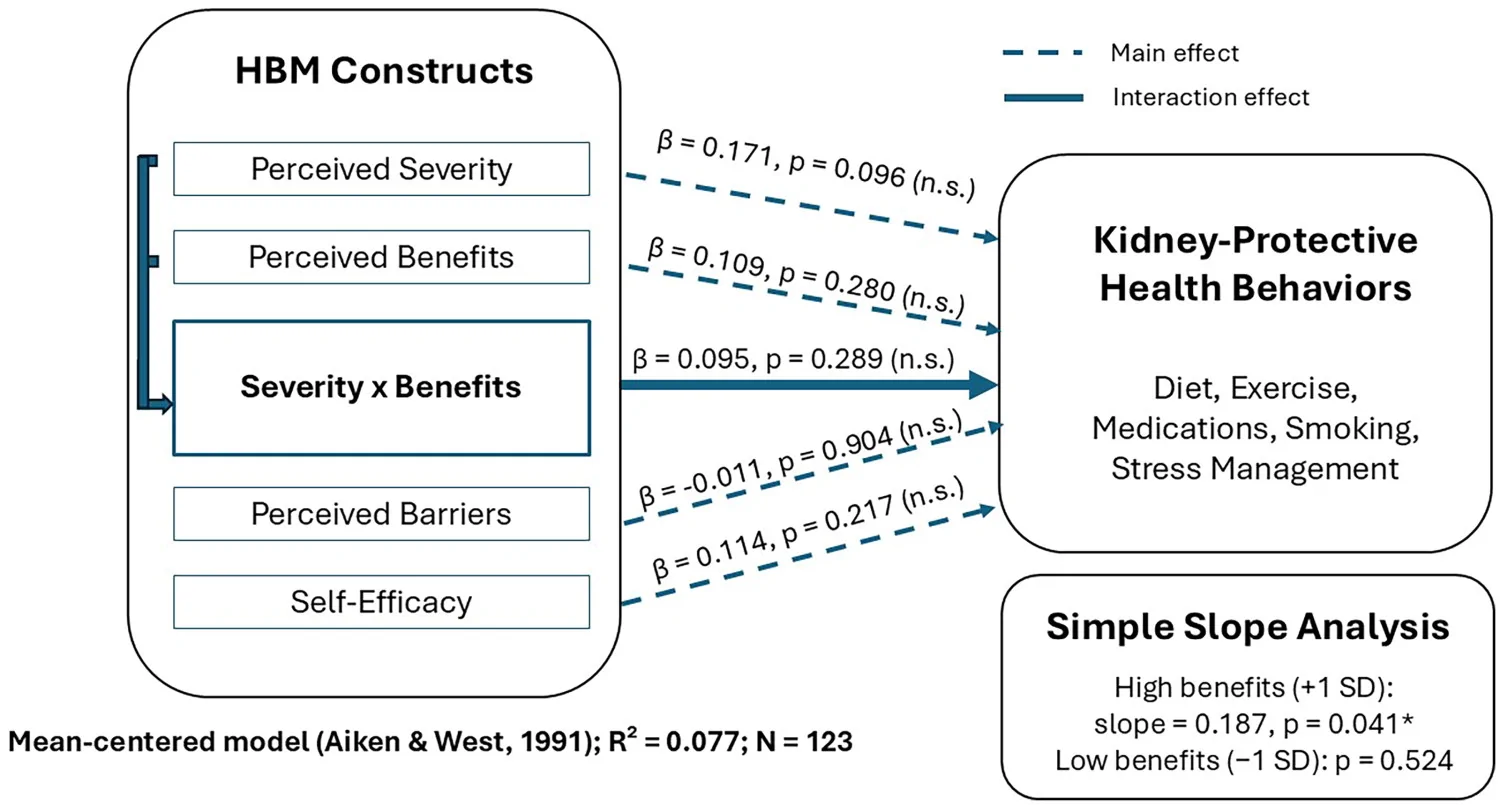
Health Belief Model (HBM) pathways predicting kidney-protective health behaviors in adults with type 2 diabetes and early kidney function decline (*n* = 123). Coefficients are from the mean-centered interaction model (Aiken & West, 1991) [23]. Dashed lines represent main effects; the solid line represents the severity × benefits interaction. No individual pathway reached statistical significance. Overall model: R^2^ = 0.077; n.s., not significant.

**Table 5.** Mean-centered interaction regression model (Aiken & West, 1991) (*N* = 123).

Predictor	b	SE	β	95% CI for β	t	*p* value	VIF
Constant	33.948	5.312	–	–	6.391	< 0.001	–
Severity (centered)	0.1245	0.0741	0.171	[−0.030, 0.371]	1.680	0.096	1.307
Benefits (centered)	0.1590	0.1466	0.109	[−0.089, 0.307]	1.085	0.280	1.278
Severity × benefits	0.0125	0.0117	0.095	[−0.081, 0.271]	1.065	0.289	1.178
Perceived barriers	−0.0156	0.1285	−0.011	[−0.193, 0.170]	−0.121	0.904	8.204
Self-efficacy	0.0686	0.0552	0.114	[−0.067, 0.294]	1.242	0.217	8.392

Note: R^2^ = 0.0772, Adjusted R^2^ = 0.0378, *F* = 1.958, *p* = 0.0900. VIF, Variance Inflation Factors.

**Table:** 

Simple slope analysis					
Level of Benefits	Simple Slope	SE	t	*p* value	95% CI
Low (−1 SD)	0.0626	0.0980	0.639	0.5243	[−0.1295, 0.2547]
Mean	0.1245	0.0741	1.680	0.0956	[−0.0207, 0.2698]
High (+1 SD)	0.1865	0.0902	2.067	0.0410*	[0.0096, 0.3633]

#### Simple slopes analysis

Simple slope analysis (Table 5; Figure 2) showed that perceived severity predicted health behaviors at high perceived benefits (+1 SD: slope = 0.187, SE = 0.090, *p* = 0.041) but not at the mean (slope = 0.125, SE = 0.074, *p* = 0.096) or at low levels (−1 SD: slope = 0.063, SE = 0.098, *p* = 0.524). The interaction term did not reach significance (*p* = 0.289); nonetheless, the slope gradient—significant only when perceived benefits were high—is directionally consistent with PMT and EPPM predictions, although these constructs were not directly measured.

**Figure 2.**
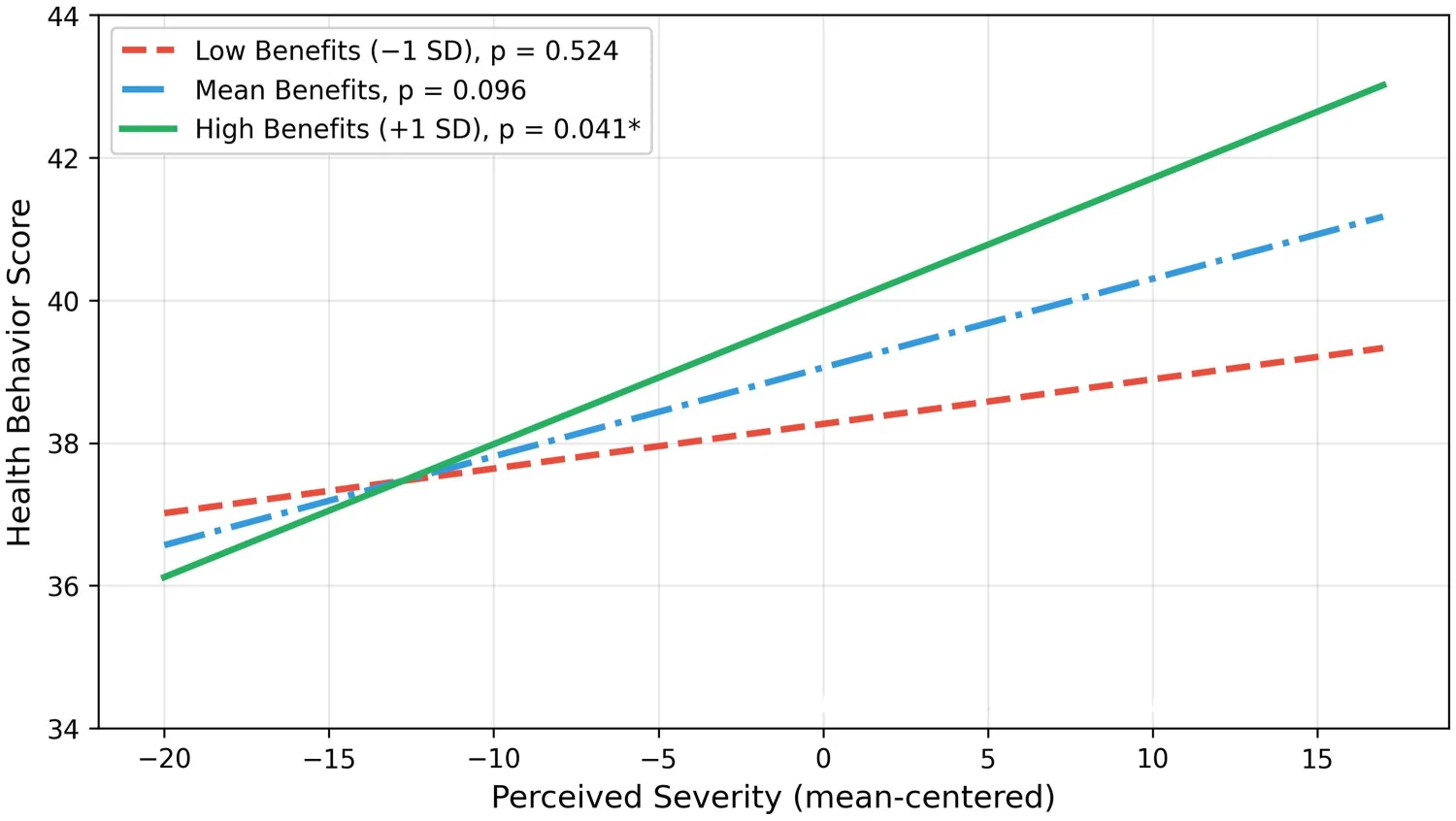
Simple slope analysis of the association between perceived severity and kidney-protective health behavior scores at high (+1 SD, green solid line), mean (blue dash-dotted line), and low (−1 SD, red dashed line) levels of perceived benefits. Perceived severity was significantly associated with health behaviors only at high perceived benefits (slope = 0.187, *p* = 0.041) but not at the mean (*p* = 0.096) or low levels (*p* = 0.524). Perceived severity values are mean-centered per Aiken & West (1991) [23].

#### Covariate-adjusted model

After adjustment for age, education, diabetes duration, HbA1c level, eGFR, and comorbidities (Table 6), the interaction pattern was maintained: the severity × benefits interaction remained the largest HBM predictor coefficient (β = 0.102, 95% CI [−0.078, 0.282], *p* = 0.265). The adjusted Model R^2^ increased modestly to 0.115, with no covariate reaching significance. This suggests that the observed pattern is not confounded by key demographic or clinical variables.

**Table 6.** Covariate-adjusted mean-centered interaction model (*N* = 123).

Predictor	b	SE	β	95% CI for β	t	*p* value
HBM Constructs						
Severity (centered)	0.0814	0.0805	0.112	[−0.106, 0.329]	1.012	0.314
Benefits (centered)	0.1667	0.1519	0.114	[−0.091, 0.319]	1.097	0.275
Severity × benefits	0.0134	0.0120	0.102	[−0.078, 0.282]	1.116	0.267
Perceived barriers	−0.0000	0.1331	−0.000	[−0.188, 0.188]	−0.000	1.000
Self-efficacy	0.0649	0.0572	0.107	[−0.080, 0.294]	1.134	0.259
Covariates						
Age	−0.0578	0.0696	−0.087	[−0.295, 0.120]	−0.830	0.408
Education (≥ bachelor)	−0.0013	1.3347	−0.000	[−0.181, 0.181]	−0.001	0.999
Diabetes duration (yrs)	0.1707	0.1260	0.136	[−0.062, 0.334]	1.355	0.178
HbA1c	0.1813	0.5394	0.034	[−0.164, 0.232]	0.336	0.737
eGFR	−0.0722	0.0545	−0.128	[−0.319, 0.063]	−1.324	0.188
Comorbidities (n)	−0.9051	1.0146	−0.085	[−0.273, 0.103]	−0.892	0.374

Note: R^2^ = 0.1148, Adjusted R^2^ = 0.0271, *F* = 1.309, *p* = 0.2288.

#### Sensitivity and exploratory analyses

A sensitivity analysis restricted to the G3a subgroup (*n* = 26) revealed directionally similar but nonsignificant results (interaction *p* = 0.619), consistent with substantially reduced power (Supplementary Table S4). Exploratory domain-specific analyses (Supplementary Table S3) revealed that no individual behavioral domain reached significance for the interaction term, with stress management showing the strongest trend (β = 0.144, *p* = 0.100). Exploratory tests for quadratic self-efficacy (*p* = 0.170) and the self-efficacy × barrier interaction (*p* = 0.686) were both nonsignificant.

## Discussion

In this cohort of Thai adults with early-stage, asymptomatic DKD, no individual HBM construct independently predicted kidney-protective behaviors. Simple slope analysis [23] revealed that perceived severity predicted health behaviors at high perceived benefits (+1 SD: *p* = 0.041) but not at low levels (−1 SD: *p* = 0.524). The interaction term did not reach significance (*p* = 0.289), likely reflecting limited power, and these exploratory findings should be regarded as hypothesis-generating rather than confirmatory evidence of moderation.

The pattern is consistent with the EPPM framework [10]: high threat combined with high efficacy promotes danger control, whereas high threat with low efficacy may provoke helplessness or avoidance rather than action. In early CKD, severity awareness alone may be insufficient without concurrent belief that self-management is worthwhile; conversely, perceived benefits without perceived urgency may lack motivational drive. Although PMT and EPPM constructs (threat appraisal, coping appraisal, fear control) were not directly measured, the gradient aligns with experimental evidence that heightened risk appraisals shift intentions and behavior principally when paired with coping-efficacy messages [24].

In contrast, perceived barriers did not predict behavior, despite commonly negative associations elsewhere [25]. Two explanations are credible: the barriers were relatively low with limited dispersion (mean 17.0/36), suggesting range restriction, and the composite behavior scale aggregates domains that may have different barrier profiles. Indeed, our exploratory domain-specific analyses revealed no significant interaction for any individual domain, which is consistent with the reduced power of the subscale analyses.

The null effect on self-efficacy warrants comment. Self-efficacy was measured with a general visual analog composite rather than domain-specific efficacy (e.g., low-sodium diet self-efficacy, exercise self-efficacy), which may have weakened the associations. Domain-specific self-efficacy measures typically show stronger associations with corresponding behavioral outcomes and would be preferable in future studies [8]. Moreover, early-stage patients may be in a primarily motivational phase rather than a volitional phase, attenuating the relative weight of self-efficacy [24]. Exploratory analyses testing a quadratic self-efficacy term (*p* = 0.170) and a self-efficacy × barriers interaction (*p* = 0.686) were both nonsignificant, suggesting that the null finding is not explained by hidden moderation or nonlinearity.

Clinically, dual-target messaging—personal risk information (eGFR trajectory, KFRE scores) paired with gain-framed communication about what patients can do and why it helps [12,24,26,27]—may translate awareness into action more effectively than severity messaging alone. In practice, this means presenting patients with their eGFR trends, blood pressure trajectories, and HbA1c values alongside concrete benefits of sodium restriction, glycemic control, medication adherence, and avoidance of nephrotoxic agents. These targets complement current diabetes-CKD pharmacotherapy and lifestyle guidelines [26]. For nursing practice in early DKD care, these findings support integrating structured dual-message counseling into outpatient self-management education: nurses and diabetes educators can pair individualized eGFR trajectory feedback with patient-specific, gain-framed behavioral goals during routine clinic encounters, reinforcing both threat awareness and actionable self-management benefits. Critically, risk information delivered without feasible, benefit-oriented self-management strategies may provoke anxiety or denial rather than protective action. These preliminary findings require confirmation in adequately powered longitudinal studies.

While the explained variance (R^2^ = 0.077) is modest, it is typical for cross-sectional studies using cognitive models alone. Health literacy, social support, psychological distress (depression, diabetes distress), socioeconomic status, access to healthcare, food environment, and habitual behavioral processes are among the determinants not captured by the HBM [11–13]. Comprehensive models integrating these factors are needed. Digitally delivered self-management platforms such as “My Kidneys & Me” [28] and DialBetesPlus [29] have shown promise in translating dual-target approaches into improved outcomes, supporting the practical feasibility of the theoretical framework [28–30].

Strengths of the study include its theory-driven design, integration of PMT/EPPM frameworks, clinically confirmed CKD staging, and proper mean-centering methodology for interaction testing [23]. Several limitations warrant emphasis. First, the sample was powered for main effects (f^2^ = 0.169) but likely underpowered for interaction detection—a probable explanation for the nonsignificant interaction term despite the significant simple slope at high benefits. Second, albuminuria data were not systematically collected, preventing definitive CKD classification for G2 participants (78.9% of the sample). Third, the cross-sectional design precludes causal inference; directionality requires longitudinal confirmation. Fourth, the single-center Bangkok setting with a relatively highly educated sample (54.5% ≥ bachelor’s degree) may limit generalizability to rural, primary care, or lower-literacy populations. Fifth, the HBM scales were developed for this study, lack factorial validation (EFA/CFA), and underwent test–retest assessment only for the VAS-based self-efficacy measure; differences in scale format between the VAS and Likert instruments may also have influenced the pattern of associations. Sixth, the composite health behavior score may have masked domain-specific relationships between HBM constructs and individual self-management behaviors. Adequately powered longitudinal or interventional studies should test whether dual-target approaches produce greater behavior change than single-target strategies.

## Conclusions

In Thai adults with type 2 diabetes and early kidney function decline, perceived severity showed a preliminary conditional association with kidney-protective behaviors only when perceived benefits were high (+1 SD: *p* = 0.041); no association was observed at low benefit levels (−1 SD: *p* = 0.524). The interaction term itself did not reach significance (*p* = 0.289), likely reflecting insufficient power, and the cross-sectional design precludes causal inference (R^2^ = 7.7%). These exploratory findings align with Protection Motivation Theory and the Extended Parallel Process Model—though neither framework was directly tested—and provide hypothesis-generating support for dual-target educational strategies combining severity awareness with benefit-oriented self-management messaging, which warrant evaluation in adequately powered longitudinal studies.

## Supplementary Material

Supplementary_Tables.docx

## Data Availability

The data that support the findings of this study are available from the corresponding author, [S.K.], upon reasonable request.
