# Supplementary material for: Perceived severity and benefits show a preliminary conditional association with kidney-protective behaviors in type 2 diabetes with early kidney function decline: an exploratory health belief model analysis
Source: Ren Fail. 2026 Oct 15;48(1):2717464. doi: 10.1080/0886022X.2026.2717464 (PMC13674644; doi:10.1080/0886022X.2026.2717464)
Supplement: Supplementary file 1 [file IRNF_A_2717464_SM1563.docx]

**Supplementary Table S1**

STROBE Statement—checklist of items that should be included in reports of observational studies

|  | | **Item No** | **Recommendation** |
| --- | --- | --- | --- |
| **Title and abstract** | | 1 | (*a*) Indicate the study’s design with a commonly used term in the title or the abstract **(P.1)** |
|  |  |  | (*b*) Provide in the abstract an informative and balanced summary of what was done and what was found **(P.3)** |
| **Introduction** | | | |
| Background/rationale | | 2 | Explain the scientific background and rationale for the investigation being reported **(PP.5-6)** |
| Objectives | | 3 | State specific objectives, including any prespecified hypotheses (**P. 6)** |
| **Methods** | | | |
| Study design | | 4 | Present key elements of study design early in the paper **(P.6)** |
| Setting | | 5 | Describe the setting, locations, and relevant dates, including periods of recruitment, exposure, follow-up, and data collection **(P.6)** |
| Participants | | 6 | (*a*) *Cohort study*—Give the eligibility criteria, and the sources and methods of selection of participants. Describe methods of follow-up  *Case-control study*—Give the eligibility criteria, and the sources and methods of case ascertainment and control selection. Give the rationale for the choice of cases and controls  *Cross-sectional study*—Give the eligibility criteria, and the sources and methods of selection of participants **(P.6-7)** |
|  |  |  | (*b*) *Cohort study*—For matched studies, give matching criteria and number of exposed and unexposed **(N/A)**  *Case-control study*—For matched studies, give matching criteria and the number of controls per case |
| Variables | | 7 | Clearly define all outcomes, exposures, predictors, potential confounders, and effect modifiers. Give diagnostic criteria, if applicable **(P.7-8)** |
| Data sources/ measurement | | 8* | For each variable of interest, give sources of data and details of methods of assessment (measurement). Describe comparability of assessment methods if there is more than one group **(PP. 7-8)** |
| Bias | | 9 | Describe any efforts to address potential sources of bias **(PP. 9)** |
| Study size | | 10 | Explain how the study size was arrived at **(P.7)** |
| Quantitative variables | | 11 | Explain how quantitative variables were handled in the analyses. If applicable, describe which groupings were chosen and why **(P.8)** |
| Statistical methods | | 12 | (*a*) Describe all statistical methods, including those used to control for confounding **(P.9)** |
|  |  |  | (*b*) Describe any methods used to examine subgroups and interactions **(N/A)** |
|  |  |  | (*c*) Explain how missing data were addressed **(N/A)** |
|  |  |  | (*d*) *Cohort study*—If applicable, explain how loss to follow-up was addressed  *Case-control study*—If applicable, explain how matching of cases and controls was addressed  *Cross-sectional study*—If applicable, describe analytical methods taking account of sampling strategy **(N/A)** |
|  |  |  | (*e*) Describe any sensitivity analyses **(N/A)** |
| **Results** | | | |
| Participants | 13* | (a) Report numbers of individuals at each stage of study—eg numbers potentially eligible, examined for eligibility, confirmed eligible, included in the study, completing follow-up, and analysed **(P.9-10)** | |
|  |  | (b) Give reasons for non-participation at each stage **(Not formally recorded; acknowledged as a reporting limitation, P.6)** | |
|  |  | (c) Consider use of a flow diagram **(N/A)** | |
| Descriptive data | 14* | (a) Give characteristics of study participants (eg demographic, clinical, social) and information on exposures and potential confounders **(PP. 10)** | |
|  |  | (b) Indicate number of participants with missing data for each variable of interest **(N/A)** | |
|  |  | (c) *Cohort study*—Summarise follow-up time (eg, average and total amount) **(N/A)** | |
| Outcome data | 15* | *Cohort study*—Report numbers of outcome events or summary measures over time **(PP. 10-11)** | |
|  |  | *Case-control study—*Report numbers in each exposure category, or summary measures of exposure | |
|  |  | *Cross-sectional study—*Report numbers of outcome events or summary measures | |
| Main results | 16 | (*a*) Give unadjusted estimates and, if applicable, confounder-adjusted estimates and their precision (eg, 95% confidence interval). Make clear which confounders were adjusted for and why they were included **(PP. 10-11)** | |
|  |  | (*b*) Report category boundaries when continuous variables were categorized **(N/A)** | |
|  |  | (*c*) If relevant, consider translating estimates of relative risk into absolute risk for a meaningful time period **(N/A)** | |
| Other analyses | 17 | Report other analyses done—eg analyses of subgroups and interactions, and sensitivity analyses **(N/A)** | |
| **Discussion** | | | |
| Key results | 18 | Summarise key results with reference to study objectives **(P.12)** | |
| Limitations | 19 | Discuss limitations of the study, taking into account sources of potential bias or imprecision. Discuss both direction and magnitude of any potential bias **(P.13-14)** | |
| Interpretation | 20 | Give a cautious overall interpretation of results considering objectives, limitations, multiplicity of analyses, results from similar studies, and other relevant evidence **(PP. 12-13)** | |
| Generalisability | 21 | Discuss the generalisability (external validity) of the study results **(P.13-14)** | |
| **Other information** | | | |
| Funding | 22 | Give the source of funding and the role of the funders for the present study and, if applicable, for the original study on which the present article is based **(P.15)** | |

*Give information separately for cases and controls in case-control studies and, if applicable, for exposed and unexposed groups in cohort and cross-sectional studies.

**Note:** An Explanation and Elaboration article discusses each checklist item and gives methodological background and published examples of transparent reporting. The STROBE checklist is best used in conjunction with this article (freely available on the Web sites of PLoS Medicine at http://www.plosmedicine.org/, Annals of Internal Medicine at http://www.annals.org/, and Epidemiology at http://www.epidem.com/). Information on the STROBE Initiative is available at www.strobe-statement.org.

**Supplementary Table S2.** Scale Items and Psychometric Properties (N = 123)

| **No.** | **English translation** | **Response format** | **Mean** | **SD** | **r**_it_ |
| --- | --- | --- | --- | --- | --- |
| **Scale 1: Perceived Severity of Diabetic Kidney Disease (10 items; 5-point Likert: 1 = strongly disagree to 5 = strongly agree; α = 0.973)** | | | | | |
| 1 | Causes easy fatigue | 5-point Likert | 3.24 | 1.25 | 0.771 |
| 2 | Reduces work capacity | 5-point Likert | 3.11 | 1.24 | 0.796 |
| 3 | Makes the heart work harder than in those without the disease | 5-point Likert | 3.33 | 1.16 | 0.774 |
| 4 | Increases the chance of developing end-stage kidney disease | 5-point Likert | 3.45 | 1.13 | 0.795 |
| 5 | Makes it impossible to live a normal life | 5-point Likert | 3.16 | 1.30 | 0.488 |
| 6 | Increases the risk of death more than in those without the disease | 5-point Likert | 3.40 | 1.19 | 0.791 |
| 7 | Requires increasingly complex treatment | 5-point Likert | 3.28 | 1.20 | 0.810 |
| 8 | Leads to higher treatment costs | 5-point Likert | 3.46 | 1.33 | 0.810 |
| 9 | Causes stress and anxiety about the severity of diabetes and CKD | 5-point Likert | 3.33 | 1.25 | 0.808 |
| 10 | Causes emotional distress | 5-point Likert | 3.33 | 1.24 | 0.767 |
| **Scale 2: Perceived Benefits of Self-Care for Slowing Kidney Deterioration (8 items; 4-point Likert: 1 = never to 4 = always; α = 0.950)** | | | | | |
| 1 | Helps maintain physical strength to perform daily activities as before | 4-point Likert | 3.15 | 0.82 | 0.654 |
| 2 | Improves one’s quality of life | 4-point Likert | 3.15 | 0.71 | 0.773 |
| 3 | Helps one live longer | 4-point Likert | 3.22 | 0.66 | 0.849 |
| 4 | Provides greater encouragement to carry on with life | 4-point Likert | 3.15 | 0.74 | 0.804 |
| 5 | Helps delay progression to end-stage kidney disease | 4-point Likert | 3.20 | 0.69 | 0.818 |
| 6 | Reduces the risk of complications from CKD | 4-point Likert | 3.15 | 0.74 | 0.822 |
| 7 | Reduces the risk of cardiovascular disease | 4-point Likert | 3.12 | 0.76 | 0.773 |
| 8 | Helps save on medical expenses | 4-point Likert | 2.99 | 0.84 | 0.759 |
| **Scale 3: Perceived Barriers to Self-Care for Slowing Kidney Deterioration (9 items; 4-point Likert: 1 = never to 4 = always a barrier; α = 0.946)** | | | | | |
| 1 | Feeling that self-care to slow kidney deterioration is difficult and burdensome | 4-point Likert | 2.07 | 0.85 | 0.553 |
| 2 | Feeling that slowing kidney deterioration is not worthwhile | 4-point Likert | 1.93 | 0.86 | 0.401 |
| 3 | Lacking necessary facilities or resources | 4-point Likert | 1.81 | 0.68 | 0.659 |
| 4 | Self-care costs to slow kidney deterioration are too high | 4-point Likert | 2.08 | 0.89 | 0.681 |
| 5 | Not having time to attend medical appointments | 4-point Likert | 1.80 | 0.86 | 0.633 |
| 6 | Having other responsibilities to attend to | 4-point Likert | 1.98 | 0.91 | 0.642 |
| 7 | Inconvenient transportation to healthcare facilities | 4-point Likert | 1.69 | 0.80 | 0.476 |
| 8 | Makes daily life more complicated | 4-point Likert | 1.92 | 0.92 | 0.589 |
| 9 | Lacking support from healthcare professionals (e.g., counseling on diet, weight control) | 4-point Likert | 1.74 | 0.90 | 0.527 |
| **Scale 4: Perceived Self-Efficacy for Slowing Kidney Deterioration (5 items; 0–100 visual analog scale; α = 0.766; ICC ≥ 0.75)** | | | | | |
| 1 | How confident are you that you can modify your diet according to medical advice? | 0–100 VAS | 74.55 | 16.56 | 0.442 |
| 2 | How confident are you that you can take medications as prescribed, on time and consistently? | 0–100 VAS | 84.39 | 13.98 | 0.482 |
| 3 | How confident are you that you can manage disease symptoms to maintain normal daily life? | 0–100 VAS | 79.43 | 15.27 | 0.594 |
| 4 | How confident are you that you can cope with problems arising during self-care for slowing kidney deterioration? | 0–100 VAS | 75.69 | 17.37 | 0.749 |
| 5 | How confident are you that you can encourage yourself when feeling stressed, anxious, or upset? | 0–100 VAS | 77.72 | 17.92 | 0.605 |
| **Scale 5: Kidney-Protective Health Behaviors (15 items; 5-point Likert: 1 = never to 5 = always; 8 reverse-scored items marked [R]; α = 0.754)** | | | | | |
| 1 | You eat foods with high fat content (e.g., fatty meat, coconut milk dishes, fried/stir-fried foods) [R] | Diet | 2.93 | 1.01 | 0.400 |
| 2 | You eat salty foods [R] | Diet | 2.43 | 0.92 | 0.286 |
| 3 | You drink tea, coffee, or carbonated beverages [R] | Diet | 3.27 | 1.50 | 0.362 |
| 4 | You exercise at moderate intensity (can still talk while exercising) | Exercise | 3.02 | 1.21 | 0.486 |
| 5 | You exercise at least 3 days per week | Exercise | 2.96 | 1.33 | 0.417 |
| 6 | You exercise at least 30 minutes per session | Exercise | 2.89 | 1.31 | 0.442 |
| 7 | You take herbal medicines [R] | Medication | 1.37 | 0.85 | 0.368 |
| 8 | You take dietary supplements [R] | Medication | 1.64 | 1.26 | 0.176 |
| 9 | You take diabetes medications as prescribed | Medication | 4.44 | 1.24 | −0.043 |
| 10 | In the past week, you smoked cigarettes [R] | Smoking | 1.14 | 0.64 | 0.183 |
| 11 | In the past week, you were exposed to secondhand smoke in public places [R] | Smoking | 1.66 | 0.92 | 0.265 |
| 12 | In the past week, you were exposed to secondhand smoke from household members [R] | Smoking | 1.27 | 0.74 | 0.329 |
| 13 | You sleep at least 6 hours per day | Stress | 3.89 | 1.14 | 0.196 |
| 14 | You manage stress through enjoyable hobbies | Stress | 3.41 | 1.17 | 0.303 |
| 15 | You manage stress by talking with a trusted person | Stress | 3.04 | 1.14 | 0.377 |

[R] = reverse-scored item (higher raw score indicates worse behavior; scored as 6 − raw score for composite calculation).

r_it_ = corrected item-total correlation (item correlated with total minus that item). Response formats: 5-point Likert (1–5); 4-point Likert (1–4); VAS (0–100).

All instruments were developed in Thai by the research team. English translations provided here are for reader reference. Content validity established by 5-member expert panel

(I-CVI: 0.70–1.00; S-CVI: 0.70–1.00). Pilot tested with 30 patients. Self-efficacy test–retest ICC ≥ 0.75.

| **Supplementary Table S3.** Domain-Specific Interaction Analyses (N = 123) | | | | | | | |
| --- | --- | --- | --- | --- | --- | --- | --- |
|  |  |  |  |  |  |  |  |
| **Domain** | **Items** | **Severity β (p)** | **Benefits β (p)** | **Interaction β (p)** | **Barriers β (p)** | **Self-Eff β (p)** | **R^2^** |
| Diet | 4 | 0.146 (0.154) | 0.126 (0.214) | 0.061 (0.494) | 0.060 (0.519) | 0.073 (0.431) | 0.0658 |
| Exercise | 2 | -0.180 (0.069) | 0.288 (0.004) | -0.044 (0.613) | 0.058 (0.515) | 0.259 (0.004) | 0.1360 |
| Medication/Herbs | 3 | 0.042 (0.686) | -0.038 (0.712) | -0.000 (0.999) | -0.004 (0.968) | 0.093 (0.327) | 0.0101 |
| Smoking | 3 | 0.294 (0.004) | -0.237 (0.017) | 0.138 (0.117) | -0.016 (0.861) | -0.076 (0.397) | 0.1107 |
| Stress Management | 3 | 0.252 (0.012) | 0.064 (0.516) | 0.144 (0.100) | -0.147 (0.104) | -0.043 (0.631) | 0.1189 |
| Composite (Total) | 15 | — | — | β = 0.095 (0.289) | — | — | 0.0772 |
| Note: All models use mean-centered interaction (Aiken & West, 1991). β = standardized coefficient. | | | | | |  |  |
| No domain-specific interaction reached statistical significance, consistent with power limitations for subscale analyses. **Supplementary: Exploratory Self-Efficacy Analyses** \| \| \|; **Analysis** \| **Coefficient** \| **p-value** \| **Interpretation**; Quadratic self-efficacy (SE^2^) \| b = -0.00407 \| 0.1701 \| No curvilinear relationship; SE × barriers interaction \| b = 0.00401 \| 0.6862 \| No moderating effect | | | | | | |  |
|  |  |  |  |  |  |  |  |

| **Supplementary Table S4.** Sensitivity Analysis: G3a Subgroup Only (n = 26) | | | | | |
| --- | --- | --- | --- | --- | --- |
|  |  |  |  |  |  |
| **Predictor** | **b** | **SE** | **t** | **p-value** |  |
| Constant | 38.5376 | 10.9311 | 3.526 | 0.0021 |  |
| Severity (centered) | 0.3328 | 0.2026 | 1.642 | 0.1161 |  |
| Benefits (centered) | -0.1686 | 0.3509 | -0.480 | 0.6362 |  |
| Severity × Benefits | -0.0214 | 0.0424 | -0.505 | 0.6193 |  |
| Perceived Barriers | 0.2156 | 0.3292 | 0.655 | 0.5200 |  |
| Self-Efficacy | -0.0255 | 0.1226 | -0.208 | 0.8373 |  |
| Note: R^2^ = 0.1763, F = 0.856, p = 0.5270 | |  |  |  |  |
| The small sample (n = 26) provides inadequate power for interaction detection. | | | | |  |
